# The Improvement of Photocatalysis H_2_ Evolution Over g-C_3_N_4_ With Na and Cyano-Group Co-modification

**DOI:** 10.3389/fchem.2019.00639

**Published:** 2019-09-19

**Authors:** Gang Liu, Song Yan, Lei Shi, Lizhu Yao

**Affiliations:** College of Chemistry, Chemical Engineering and Environmental Engineering, Liaoning Shihua University, Fushun, China

**Keywords:** graphitic carbon nitride, Na and cyano-group co-modification, photocatalysis, H_2_ evolution, sustainable

## Abstract

Na and cyano-group co-modified g-C_3_N_4_ was easily synthesized and its physicochemical property was completely analyzed. The results manifested that Na and cyano-group modification could heighten visible light absorbed ability and accelerate photoinduced charge separation. When resultant Na and cyano-group co-modified g-C_3_N_4_ was splitting water H_2_ evolution, its H_2_ evolution rate was obviously improved. Furthermore, it also kept excellent stable capacity of H_2_ evolution and stability of chemical structure. Hence, this present study does not only develop an efficient strategy to boost photocatalytic property of g-C_3_N_4_ based catalysts, but also provides useful guidance for designing more effective photocatalysts.

## Introduction

In past decades, with excessive consumption of fossil energy (petroleum, coal, natural gas, etc.), human beings are facing enormous challenge in energy shortage. Hence, the utilization of new energy has been a hot topic. Among them, as a reproducible energy, solar energy exhibits some merits, such as generalization, abundance, clean. As a result, as one of methods for using solar energy, photocatalytic technology has attracted wide attention. All kinds of photocatalysts (TiO_2_, ZnO, SnO_2_, etc.) have been prepared and applied in photocatalytic filed (Chen and Mao, 2007; Shekofteh-Gohari et al., 2018). However, to adequately use solar energy, the development of more kind photocatalysts is of significance.

Recently, due to narrow band gap, low cost and easy preparation, graphitic carbon nitride (g-C_3_N_4_) has gained worldwide view (Wang et al., 2009; Liu et al., 2015). Whereas, unmodified g-C_3_N_4_ has many unfavorable factors, such as easy combination of photoinduced electrons and holes. It is difficult to meet the needs of industrialization. Therefore, to gain the improved photocatalytic performance, some strategies have been employed for modifying g-C_3_N_4_. One effective strategy was doped metal (Chen et al., 2009; Sun et al., 2017) or non-metal (Zhou et al., 2015; Li et al., 2016; Thaweesak et al., 2017). The separation rate of photogenerated charge could be effectively enhanced by this method. Other strategies were also applied for boosting photocatalytic performance of the g-C_3_N_4_. Zheng et al. produced hollow-like g-C_3_N_4_, its photocatalytic performance was improved via increasing specific surface area and enhancing light absorption (Zheng et al., 2016). Lin et al. modified the crystallinity and polymerization degree of g-C_3_N_4_ to improve photocatalytic property (Lin et al., 2018). Zhang et al. established isotype heterojunction to facilitate photogenerated charge separation, which could improve the efficiency of visible light photocatalytic splitting water to produce hydrogen (Zhang et al., 2012). And other g-C_3_N_4_ based heterojunctions, including BiOBr/g-C_3_N_4_ (Ye et al., 2013), g-C_3_N_4_/Bi_2_WO_6_ (Ge et al., 2011), MoS_2_/g-C_3_N_4_ (Ge et al., 2013), g-C_3_N_4_/TiO_2_ (Huang et al., 2015), ZnO/g-C_3_N_4_ (He et al., 2015), In_2_O_3_/g-C_3_N_4_ (Cao et al., 2014), FeOx/g-C_3_N_4_ (Cheng et al., 2016), were successfully established. Obviously, a great many of techniques were exploited to boost photocatalytic property of g-C_3_N_4_. From practical application perspective, the more modified methods are developed, the large-scale application is the simpler.

Therefore, in this paper, Na and cyano-group co-modified g-C_3_N_4_ was successfully synthesized. The results manifested that Na and cyano-group modification could heighten visible light absorbed ability and accelerate the photoinduced charge separation, when resulted Na and cyano-group co-modified g-C_3_N_4_ was applied for splitting water H_2_ evolution, its H_2_ evolution rate obviously higher than previous g-C_3_N_4_. Furthermore, it also kept excellent stable capacity of H_2_ evolution and stability of chemical structure. Hence, this present study does not only develop an effective strategy to boost the photocatalytic property of g-C_3_N_4_, but also supplies useful guidance for designing efficient photocatalysts.

## Experimental

### Synthesis of Catalysts

Melamine (Analytical Grade) and NaOH (Analytical Grade) were purchased in Sinopharm Chemical Reagent Co. Ltd, China. Deionized water was used in this study.

g-C_3_N_4_ was obtained by thermopolymerization of melamine (10 g) at 550°C for 120 min in a covered crucible under the air, and denoted as MCN. Na and cyano-group co-modified g-C_3_N_4_ was synthesized by following step: MCN (0.5 g) and NaOH (0.1 g) were grinding for 10 min. Resulting hybrid was heated at 450°C for 120 min under N_2_ atmosphere in tube furnace. After the tube furnace cool down, the product was washed by enough deionized water and ethanol and dried. The product was denoted as Na-CMCN.

### Characterizations and Photocatalytic Experiments

The detail was supplied in Supporting Information.

## Results and Discussion

**Figure 1:**
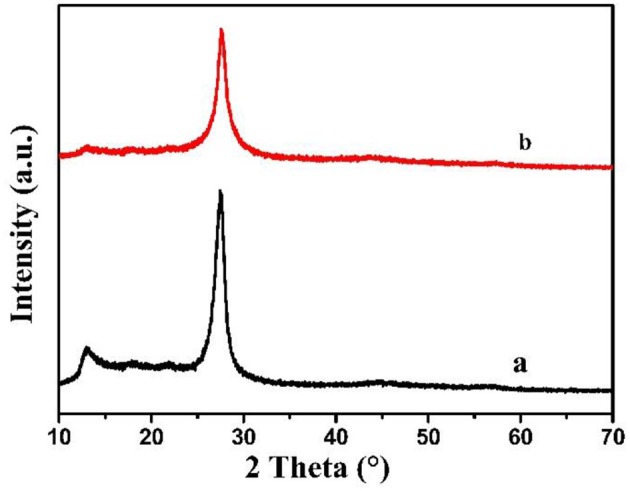
The XRD patterns of (a) MCN and (b) Na-CMCN.

In Figure 1, the XRD patterns of MCN and Na-CMCN were characterized. Both of them exhibited two peaks (13.1 and 27.4°). The stronger peak belonged to (002) peak, resulted from the interlayer stack of g-C_3_N_4_ sheets (Wang et al., 2010). The weak peak belonged to (100) peak, caused by in-plane packing of g-C_3_N_4_. By comparison, two peaks of Na-CMCN become weaker than MCN, demonstrating that g-C_3_N_4_-basic structure might be destroyed at a certain extent due to the introduction of NaOH.

**Figure 2:**
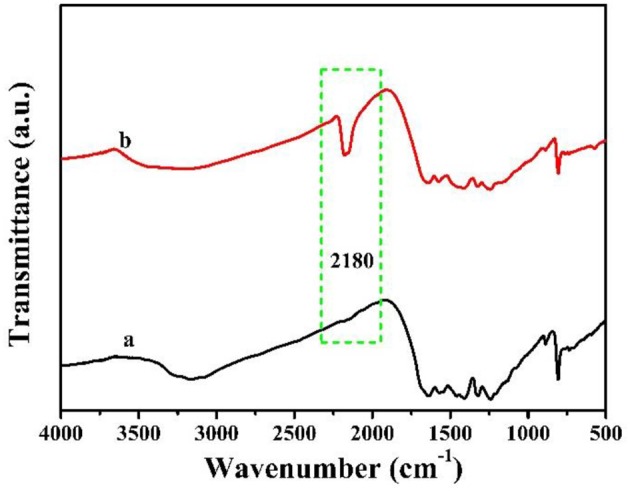
The FTIR spectra (a) MCN and (b) Na-CMCN.

The FTIR of as-prepared MCN and Na-CMCN were showed in Figure 2. The typical intensity peak of all samples showed at 808, 1,100–1,600, and 3,000–3,500 cm^−1^ three sections, respectively. The structure of heptazine rings was showed at the first section (Fang et al., 2017). The second section demonstrated the CN heterocycle and the –NH_2_ and –OH were reflected at the final section (Dante et al., 2011). Compared with MCN, 2,180 cm^−1^ for Na-CMCN could be found, which belonged to the cyano groups (C=N) (Liang et al., 2018; Yuan et al., 2018). This result meant that cyano groups could be introduced in g-C_3_N_4_-basic structure during the post-treatment at the existence of NaOH.

**Figure 3:**
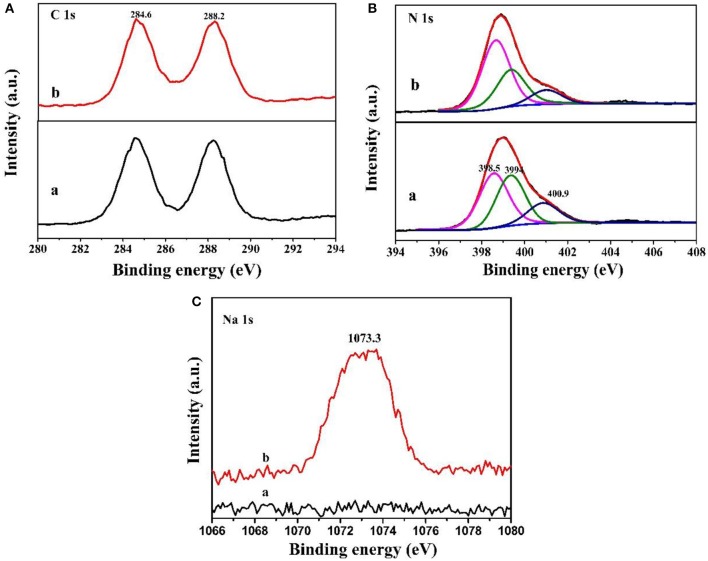
The XPS spectra of **(A)** C 1s, **(B)** N 1s, and **(C)** Na 1s in (a) MCN and (b) Na-CMCN.

In addition, the surface chemical composition of MCN and Na-CMCN was measured by XPS in Figure 3. In C 1s spectrum (Figure 3A), 284.6 and 288.2 eV two peaks could be found. The first peak belonged to the outside elements of C, the last peak ascribed to N-C = N (Liang et al., 2019a). In addition, three components were exhibited in N 1s spectra at 400.9, 399.4, and 398.5 eV in Figure 3B, corresponding to the N-H or N-H_2_, the tertiary nitrogen and the sp^2^ hybridized CN in the heptazine rings, respectively (Chen et al., 2017; Wang et al., 2019). Interestingly, in Figure 3C, the peak was detected in the high-resolution Na 1s spectrum over Na-CMCN, but no peak was found in MCN, which denoted that Na element was successfully doped in the Na-CMCN basic structure. Furthermore, the Na spectra of Na-CMCN located at 1073.3 eV, which indicated that Na^+^ ions was doped in the g-C_3_N_4_ through Na-N bond (Sudrajat, 2018; Tripathi and Narayanan, 2019).

**Figure 4:**
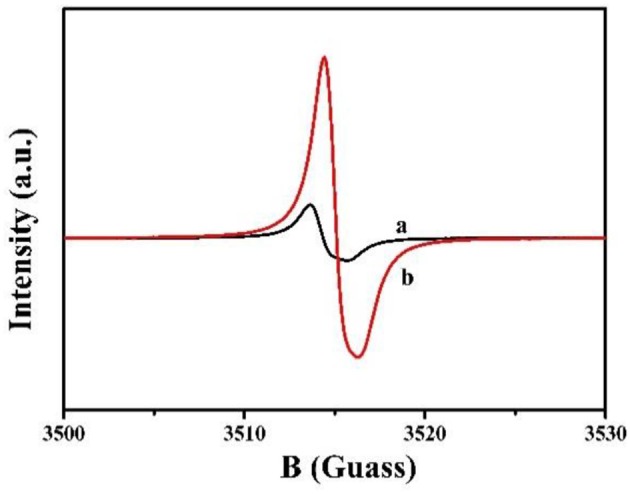
Room-temperature EPR spectra (a) MCN and (b) Na-CMCN.

The electronic band structure for MCN and Na-CMCN was measured by electron paramagnetic resonance spectra (EPR) in Figure 4. Compared with MCN, the EPR signal intensity of Na-CMCN has clearly increased. This phenomenon indicated that more unpaired electrons were presented in the localized heterocyclic ring of Na-CMCN (Zhang and Wang, 2013).

**Figure 5:**
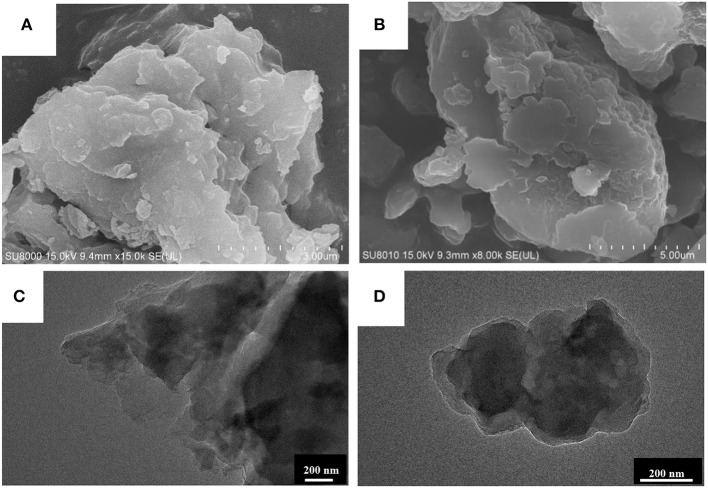
The SEM images of **(A)** MCN and **(B)** Na-CMCN and TEM images of **(C)** MCN and **(D)** Na-CMCN.

The morphologies of the MCN and Na-CMCN were surveyed using SEM and TEM. MCN exhibited block structure in Figures 5A,C. For Na-CMCN, in Figure 5B, the part of block structure was desquamated. In Figure 5D, Na-CMCN presented block structure, besides several pores formed on its surface, which might be due to the NaOH corrosion.

**Figure 6:**
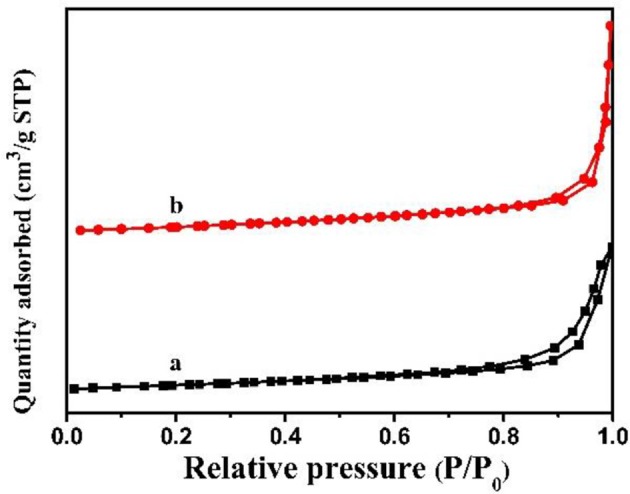
N_2_ adsorption-desorption isotherms of (a) MCN and (b) Na-CMCN.

In addition, N_2_ adsorption-desorption isotherms of the MCN and Na-CMCN were measured in Figure 6. Two photocatalysts presented similar IV N_2_ adsorption isotherms. The BET surface areas of MCN and Na-CMCN were 9.7 and 7.6 m^2^ g^−1^, respectively. There was not evident change for their surface areas.

**Figure 7:**
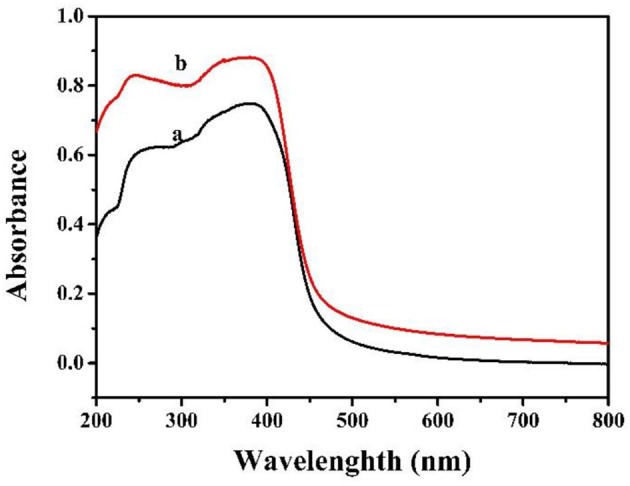
The DRS of (a) MCN and (b) Na-CMCN.

Figure 7 demonstrates the light absorption of MCN and Na-CMCN. MCN and Na-CMCN presented the similar absorb edge, the main difference was that Na-CMCN possessed enhanced light absorption in comparison to MCN between 400 and 800 nm, which indicated that the improvement of visible light photocatalytic property over Na-CMCN might be anticipated.

**Figure 8:**
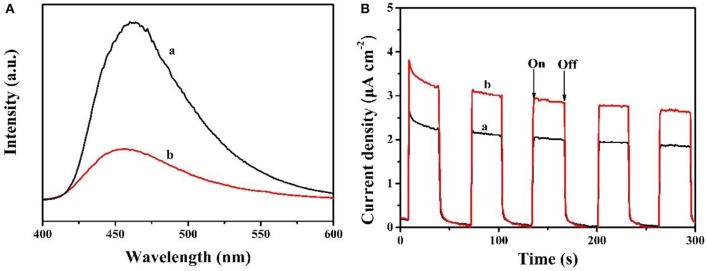
**(A)** Photoluminescence curves and **(B)** Transient photocurrent curves of (a) MCN and (b) Na-CMCN.

The separation rate of photoinduced charge carriers for MCN and Na-CMCN were performed on photoluminescence (PL) curves in Figure 8A. Na-CMCN sample indicated the lower PL signal in comparison to MCN sample. The decreased peak intensity was resulted from the separation of the charge carries (Yu et al., 2014; Liang et al., 2019b). Evidently, Na-CMCN exhibited better separation effect of photoinduced charge. Furthermore, transient photocurrent responses of MCN and Na-CMCN were measure and shown in Figure 8B. A stable photocurrent response was demonstrated in each on and off cycle. The current density of the Na-CMCN was higher than that of the MCN, which indicates that the Na-CMCN had better separation ability of photoinduced charge (Tian et al., 2019). The result firmly confirmed the result of PL spectra.

**Figure 9:**
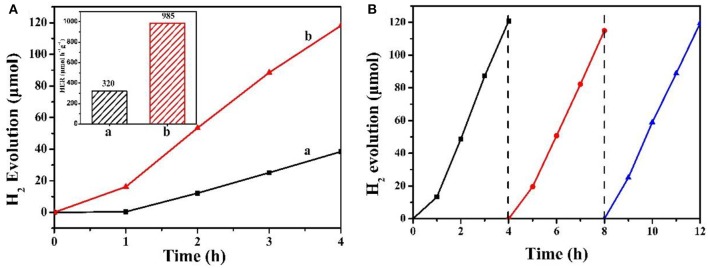
**(A)** Photocatalytic H_2_ evolution rate of (a) MCN and (b) Na-CMCN (Inset is HRE). **(B)** Stability test for H_2_ photosynthesis over Na-CMCN.

Then, the photocatalytic performance of as-prepared MCN and Na-CMCN was investigated. Figure 9A presents the photocatalytic capacity for splitting water H_2_ evolution over both of photocatalysts. With the extension of the illumination time, H_2_ was continuous produced. Obviously, Na-CMCN showed better performance for H_2_ evolution in the same time. Whereafter, to further compared their photocatalytic activity, Inset shows H_2_ evolution rate (HER) of both of photocatalysts. Obviously, Na-CMCN emerged higher activity for H_2_ evolution in the same time. Its HER was 3.07-folds more than MCN (985 vs. 320). Not only that, a recycled experiment for H_2_ evolution over as-prepared Na-CMCN was also measured. As shown in Figure 9B, after 3 times recycled experiments, Na-CMCN exhibited no significant deactivation, indicating that as-prepared Na-CMCN is an effective and steady visible light photocatalyst.

**Figure 10:**
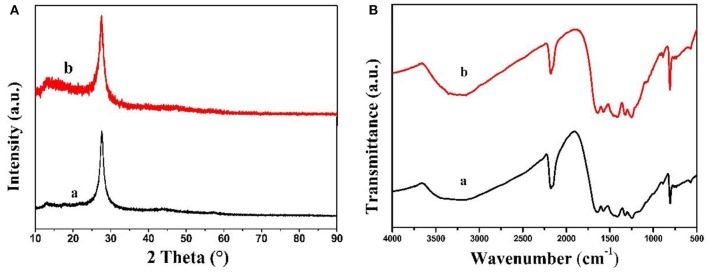
**(A)** XRD patterns and **(B)** FTIR of (a) fresh Na-CMCN and (b) used Na-CMCN.

In addition, reacted Na-CMCN was analyzed using XRD and FTIR. The results were presented in Figure 10. By comparison, two distinct XRD diffraction peaks and infrared representative stretching vibration modes of fresh Na-CMCN and used Na-CMCN were almost changeless, implying that chemical structure of Na-CMCN maintained very steady.

## Conclusion

In this paper, a facile and successful method was used to prepare Na and cyano-group co-modified g-C_3_N_4_ (Na-CMCN). The results found that Na and cyano-group modification could heighten visible light absorb ability and accelerate photoinduced charge separation, resulting that Na-CMCN exhibited higher HER than previous g-C_3_N_4_ (985 vs. 320). Not only that, it also maintained excellent stable capacity of H_2_ evolution and stability of chemical structure. As a result, this present study does not only develop an effective strategy to boost photocatalytic property of g-C_3_N_4_, but also supplies useful guidance for projecting efficient photocatalysts.

## Data Availability Statement

All datasets generated for this study are included in the manuscript/Supplementary Files.

## Author Contributions

GL: measurement of performance. LS: design of catalyst. SY and LY: characterization of catalyst.

### Conflict of Interest

The authors declare that the research was conducted in the absence of any commercial or financial relationships that could be construed as a potential conflict of interest.
