# Supplementary material for: The Improvement of Photocatalysis H2 Evolution Over g-C3N4 With Na and Cyano-Group Co-modification
Source: Front Chem. 2019 Sep 19;7:639. doi: 10.3389/fchem.2019.00639 (PMC6761803; doi:10.3389/fchem.2019.00639)
Supplement: Supplementary file 1 [file Table_1.docx]

**The improvement of photocatalysis H_2_ evolution over g-C_3_N_4_ with Na and cyano-group co-modification**

Gang Liu ^a^, Song Yan ^a^, Lei Shi ^a^*, Lizhu Yao ^a^

a: College of Chemistry, Chemical Engineering and Environmental Engineering, Liaoning Shihua University, Fushun 113001, China

*Corresponding author: Lei Shi, [shilei_hit@qq.com](mailto:shilei_hit@qq.com)

Tel: +86-02456861842

***Characterization***

X-ray diffraction pattern (XRD) was used for detecting phase identification of samples on a Bruker D8 Advance X-ray powder diffractometer. Fourier transform infrared spectroscopy (FTIR) of samples were collected using KBr discs on Perkin Elmer spectrum 100. X-ray photoelectron spectroscopy (XPS) was carried out to analyze the chemical state and composition on Thermo Fisher Scientific Escalab 250 using a spectrometer with a monochromatized Al Ka X-ray source and the shift of the binding energy was calibrated using an internal standard of C1s level at 284.6 eV. Then the relate element XPS spectra was fitted using the XPS PEAK 4.1. Electron paramagnetic resonance (EPR) was measured on a Bruker EMX-10/12 EPR spectrometer. The morphologies and microtopographies of samples were also recorded by scanning electron microscopy (SEM, SU8010, Hitachi) and transmission electron microscopy (TEM, JEM-2010), respectively. N_2_ adsorption-desorption isotherms were collected at 77 K on a Quantachrome NOVA 2000 surface area and porosity analyzer. The UV–vis diffuse reflectance spectra (DRS) of samples were measured on Agilent Cary 5000 UV–vis spectrometer. The photoluminescence spectra (PL) were recorded with an excitation wavelength of 325 nm on Agilent Cary Eclipse spectrometer. Transient photocurrent response of the samples were obtained by using a CHI760 electrochemical system (Shanghai, China). 300W Xe lamp with a UV-cutoff filter supplied light source, and 0.1 M Na_2_SO_4_ was used as the electrolyte solution.

***Photocatalytic testing***

The visible-light-induced catalytic H_2_ evolution was carried out in a Pyrex top-irradiation reaction vessel connected to a sealed glass gas-circulation system. 0.030 g sample was dispersed in an aqueous solution containing deionized water (45 ml) and triethanolamine (5ml). Pt as co-catalyst (3 wt%) was in situ photo-deposited on the catalyst by using H_2_PtCl_6_. And the reaction system was kept at about 6 ^o^C with circulating cooling water. Prior to visible light irradiation, the air in reactant solution was evacuated repeatedly by vacuum pump, followed by irradiation with a 300 W Xenon lamp equipped with a UV-cutoff filter (λ>400 nm) and 15 A working current. As photocatalysis reaction proceeded, the amount of H_2_ was analyzed every 1 h by online gas chromatography equipped with a thermal conductivity detector and Ar was used as the carrier gas.
